# Prophylaxis with enoxaparin and antithrombin III in drug-induced coagulation alterations in childhood leukemia: a retrospective experience of 20 years

**DOI:** 10.1186/s12959-024-00602-x

**Published:** 2024-03-27

**Authors:** Christina Salvador, Robert Salvador, Gabriele Kropshofer, Bernhard Meister, Marie Rock, Petra Obexer, Benjamin Hetzer, Evelyn Rabensteiner, Roman Crazzolara

**Affiliations:** 1 Department of Pediatrics I, Division of Hematology and Oncology, Medical University of Innsbruck, Anichstr. 35, Innsbruck, 6020 Austria; 2 HTLinn, Innsbruck, Austria; 3 Department of Pediatrics II, Medical University of Innsbruck, Innsbruck, Austria

**Keywords:** Antithrombin III, Children, Coagulation, Enoxaparin, L-asparaginase, Leukemia

## Abstract

**Background:**

Thromboembolic complications are well known in the treatment of childhood acute lymphoblastic leukemia. Over the years it has not been possible to reach a consensus on a possible prophylaxis of thromboembolic events during intensive therapy. Only the administration of enoxaparin was able to achieve evidence in the literature to date.

**Methods:**

In this retrospective study, 173 childhood leukemia patients were treated over 20 years with a thromboembolic prophylaxis including enoxaparin and AT III during induction therapy with L-asparaginase and cortisone.

**Results:**

We here report the effectiveness of administration of enoxaparin and AT III in childhood leukemia, showing a strikingly low prevalence of deep vein thrombosis (2.9%). Especially in adolescent patients, a particularly great need for AT III was demonstrated.

**Conclusions:**

We recommend thromboembolic prophylaxis with enoxaparin and AT III substitution during induction/reinduction therapy with L-asparaginase and glucocorticosteroids, especially from adolescence onwards.

## Introduction

Thromboembolic complications are frequently observed during induction therapy of childhood acute lymphoblastic leukemia (ALL) [1]. They can significantly increase morbidity of this malignant disease and often lead to long-term consequences. Potential reasons for this increased tendency to thrombosis could, on the one hand, be the disease itself, on the other hand treatment with chemotherapeutic agents, in particular L-asparaginase (ASP) and glucocorticoids [2]. L-asparaginase is widely used in the treatment of pediatric ALL. Besides allergic reactions, therapy with ASP is associated with coagulopathy due to a decrease in almost all proteins responsible for coagulation and anticoagulation, but antithrombin III (AT III) is affected to the greatest extent [3]. Early detection and prevention of thromboembolic complications during ALL induction is important to reduce potentially life-threatening events and ensure timely continuation of therapy. Thus, several authors agree that prophylaxis with anticoagulants should be considered in this sensitive phase of therapy [4]. However, to date there is no generally valid consensus on the effectiveness of thromboembolic prophylaxis in children with cancer and different centers take different approaches [5]. As early as 2008, we were able to show that the use of prophylactic low-molecular-weight heparin (LMWH) in combination with AT III substitution led to a significant reduction in severe thrombosis without increasing the risk of major bleeding [6]. More recent recommendations in the literature support the claim that LMWH can effectively and safely reduce thromboembolic complications in childhood ALL [7]. There is a need for large, prospective studies that compare different approaches with each other so that a statement can also be made about other strategies such as use of vitamin K antagonists, AT III replacement or other options. In a prospective study, Greiner et al. recommended thromboprophylaxis with enoxaparin (one of the most common LMWH) in children and adolescents with ALL [8]. Another randomized controlled trial is currently underway on thromboprophylaxis with LMWH in childhood leukemia [9]. Newer anticoagulants such as apixaban are also currently being tested in randomized studies for their effectiveness in reducing thrombosis in children with ALL [10].

However, in addition to these certainly very important prospective studies, many years of clinical experience with successful management of thrombosis prophylaxis in childhood ALL can produce an important statement regarding the effectiveness and safety of these procedures. In the following retrospective analysis, we describe 20 years of experience with strikingly successful thromboembolic prophylaxis with enoxaparin plus AT III substitution during ALL induction therapy.

## Material and methods

### Patients

The retrospectively evaluated principal cohort included all children and adolescents (age > 1 year to < 18 years) with newly diagnosed ALL at our institution (Department of Pediatrics I, Medical University of Innsbruck) between June 2001 and July 2021, included in the randomized controlled trials ALL-BFM 2000, AIEOP-BFM ALL 2009 or 2017. Originally, 97 patients from the ALL-BFM 2000, 94 patients from the AIEOP-BFM ALL 2009, and 26 patients from the AIEOP-BFM ALL 2017 study were evaluated. However, due to lack of data sets or lack of comparability in the case of randomization with increased L-asparaginase administration (ASP +), 25 patients from ALL-BFM 2000, 16 patients from AIEOP-BFM ALL 2009, and three patients from AIEOP-BFM ALL 2017 were excluded. Thus, the effective study cohort included 173 patients, 74 (42.8%) of them female and 99 (57.2%) male. Mean age at diagnosis was 6.66 years (± 4.49 standard deviation; range 0.22 – 17.92 years; Table 1). All patients received chemotherapy according to the AIEOP-BFM ALL study protocols (2000, 2009 or 2017), including prednisone 60 mg/m^2^/d (7 days prephase, 21 days normal dose, tapering; AIEOP-BFM 2000: randomization prednisone versus dexamethasone), vincristine 1.5 mg/m^2^/d (days 8, 15, 22, 29), daunorubicin 30 mg/m^2^/d (days 8, 15, 22, 29), E.coli asparaginase 5000 IU/m^2^/d for ALL-BFM 2000 (8 times during induction from day 12) or PEG L-asparaginase 2500 IU/m^2^/d for AIEOP-BFM ALL 2009/2017 (days 12 and 26). Prior to chemotherapy, a central catheter (Broviac/Hickman) was implanted in the subclavian vein of each patient. Relevant for this study is the comparable induction phase in all three studies with i.v. administration of asparaginase (eight times E. coli asparaginase for ALL-BFM 2000 and twice PEG L-asparaginase for AIEOP-BFM ALL 2009/2017) and identical cortisone therapy. All patients included in this retrospective evaluation received daily prophylaxis with enoxaparin subcutaneously from the day of first asparaginase administration (dose 1 mg/kg) to normalization of coagulation parameters. Antithrombin III (AT III) was additionally substituted intravenously below a limit of 50%. This prophylactic combination therapy with enoxaparin and AT III was discontinued for platelets below 30G/L, but was otherwise administered until the coagulation values normalized (especially AT III levels above 50% without substitution and fibrinogen stable above 100 mg/dL). The diagnosis of deep vein thrombosis was made by ultrasound in all cases, except in the patient who developed sinus vein thrombosis, where the diagnosis was made using computed tomography.

**Table 1:** Patient characteristics

	**ALL-BFM 2000**	**ALL-BFM 2009**	**ALL-BFM 2017**	**Total population**
**Number of patients**	72	78	23	173
**Sex**				
Female	35	30	9	74 (42.8%)
Male	37	48	14	99 (57.2%)
**Age groups**				
< 10 years of age	52	65	21	138
> 10 years of age	20	13	2	35
**Age at diagnosis** **[years ± standard deviation]**	7.28 ± 4.64	6.28 ± 4.69	6.05 ± 3.01	6.66 ± 4.49

All our patients undergo a basic assessment of coagulation parameters at the start of intensive therapy.

### Laboratory methods

Routine blood specimens for coagulation parameters were drawn at least twice a week during induction therapy (first 40 days). If abnormalities such as a reduced AT III level were detected, blood specimens were drawn daily to ensure timely substitution. AT III was determined using a chromogenic substrate from citrated plasma in our central laboratory (Innsbruck Medical University Hospital). Substitution of AT III was performed below a level of 50% according to the following formula: required units = body weight [kg] x (100 – current antithrombin activity [%]) × 2/3.

### Statistical analysis

Study data were analyzed with descriptive and inferential statistics using Python as programming language along with several special Python modules (pandas, numpy, scipy). Statistical differences of mean values were calculated by means of customary T tests – with preceding F tests, as usually required. Resulting *p* values and confidence intervals are based on a confidence level of 95%. Consequently, *p* values below α = 0.05 are deemed statistically significant. Confidence intervals of regression slopes or mean value differences that are completely located in the positive or negative range are considered statistically significant as well as corresponding with *p* values below 0.05.

### Ethics

The Ethics Committee of the Medical University of Innsbruck approved retrospective evaluation (EC No. 1478/2020). All data were obtained from medical records. This study was performed in accordance with the Declaration of Helsinki.

## Results

### Decline in coagulation parameters during induction therapy

**Fig. 1:**
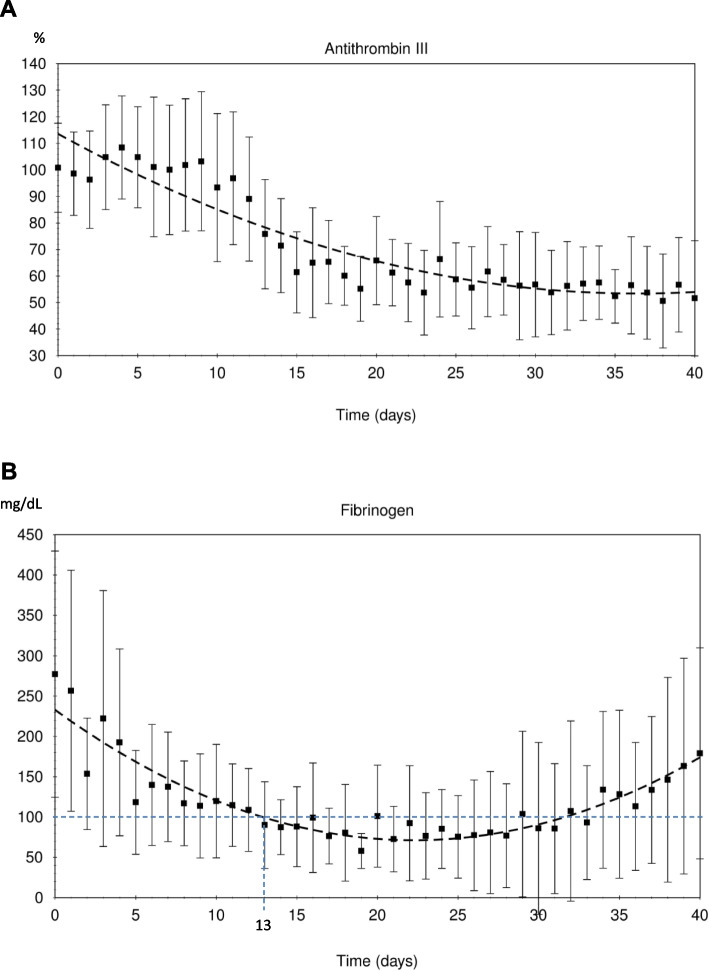
**A** Decrease in antithrombin III during induction therapy. Substitution was performed at less than 50% serum level. (expressed as mean values with standard deviation and visualized as regression curve). **B** Decrease in fibrinogen during induction therapy. Levels dropped below 100 mg/dL at day 13 of induction therapy (expressed as mean values with standard deviation and visualized as regression curve)

As has already been shown several times in the literature, we, too, can demonstrate a significant drop in the relevant coagulation parameters during therapy with asparaginase and cortisone in our ALL cohort. In the process, fibrinogen dropped below a level of 100 mg/dL at day 13 of induction therapy (Fig. 1B; regression curve based on the mean values of the measured fibrinogen). AT III was continuously substituted in our patient cohort for values below 50%. Therefore, the drop observed here is not as sharp as described in the literature and the minimum mean values in our figure settle at about 50% (Fig. 1A).

### Prophylactic combination therapy with enoxaparin and antithrombin III during induction therapy

Prophylaxis with enoxaparin was performed in 167 of the 173 patients in the ALL cohort examined, i.e. the compliance for this supportive measure was very high as compared to other studies that administered this therapy [8] (96.5%; Table 2). The 6/173 (3.5%) patients who did not receive enoxaparin are all in the ALL 2000 study, which could possibly be due to the moderate experience with this prophylaxis at that time and the lack of staff training. Substitution of AT III was performed in 122 (70.5%) of the 173 patients; only 51 of the 173 patients did not receive any AT III (values always measured above 50%, 29.5%; Table 2). In the 122 patients substituted with AT III, we were able to evaluate a total of 173 AT III doses. The frequency of doses showed a mean value of 2.06 doses per patient during the induction phase of therapy (median 2.0; standard deviation ± 2.0; range 0—9 doses). There was no significant difference between the sexes or the frequency of AT III administration (*p* = 0.076; mean doses female/male: 1.76/2.28; range female/male: 0–6 versus 0–9). Duration of the required AT III substitutions was on average 7.84 days after start of AT III substitution (range 0–45 days). Again, there was no significant difference in gender (*p* = 0.248; mean value female/male: 6.9/8.56 days). In terms of age, we found a high statistical significance regarding the need for AT III substitution (total population *p* < 0.0001; female *p* < 0.0001; male *p* = 0.003). This is also demonstrated in Table 2: In the age group of patients < 10 years of age, 65.2% required AT III substitution, whereas in the age group > 10 years of age 91.4% of patients needed AT III administration, calculated for the total population (Table 2). Likewise, the duration of the necessary AT III substitution in female patients was statistically significantly dependent on age (total population *p* = 0.064; female *p* = 0.022; male *p* = 0.567). That means adolescent patients have a greater need for AT III substitution, and female adolescent patients need these substitutions for a longer time.

**Table 2:** Summary of enoxaparin prophylaxis and AT III administration and the frequency of thrombotic events

	**ALL-BFM 2000**	**ALL-BFM 2009**	**ALL-BFM 2017**	**Total population**
**Enoxaparin prophylaxis during L-asparaginase**				
Yes	66/72 (91.7%)	78/78 (100%)	23/23 (100%)	167/173 (96.5%)
No	6/72 (8.3%)	0/78 (0%)	0/23 (0%)	6/173 (3.5%)
**ATIII substitution during enoxaparin prophylaxis**				
**Yes**	48/72 (66.7%)	56/78 (71.8%)	18/23 (78.3%)	122/173 (70.5%)
< 10 years of age	29/52 (55.8%)	45/65 (69.2%)	16/21 (76.2%)	90/138 (65.2%)
> 10 years of age	19/20 (95%)	11/13 (84.6%)	2/2 (100%)	32/35 (91.4%)
**No**	24/72 (33.3%)	22/78 (28.2%)	5/23 (21.7%)	51/173 (29.5%)
**Number of ATIII administrations**				
0 times	24/72 (33.3%)	22/78 (28.2%)	5/23 (21.7%)	51/173 (29.5%)
1 time	16/72 (22.2%)	13/78 (16.7%)	3/23 (13.0%)	32/173 (18.5%)
2 times	9/72 (12.5%)	14/78 (18.0%)	5/23 (21.7%)	28/173 (16.2%)
3 times	8/72 (11.1%)	14/78 (18.0%)	5/23 (21.7%)	27/173 (15.6%)
4 or more times (max. 9 times)	15/72 (20.8%)	15/78 (19.2%)	5/23 (21.7%)	35/173 (20.2%)
**Blockade of central venous catheter during induction**	7/72 (9.7%)	4/78 (5.1%)	3/23 (13.0%)	14/173 (8.1%)
**Deep vein thrombosis**				
Yes	3/72 (4.2%)	1/78 (1.3%)	1/23 (4.3%)	5/173 (2.9%)
No	69/72 (95.8%)	77/78 (98.7%)	22/23 (95.7%)	168/173 (97.1%)

### Very low incidence of thromboembolic complications under prophylaxis with enoxaparin and AT III

We analyzed the incidence of both mild thromboembolic complications (e.g. blockage of the central venous catheter during induction therapy) and severe deep thrombosis and came to the conclusion that the incidence under prophylaxis with enoxaparin and AT III is significantly lower than in the described literature:

Blockade of central venous catheter during induction therapy (first 50 days) was seen in 14 of the 173 patients (8.1%; Table 2) who needed lysis. Deep vein thrombosis was observed in only five of the 173 patients (2.9%) in our ALL cohort (Table 2; 3/72 patients (4.2%) in the ALL-BFM 2000 study, 1/78 patients (1.3%) in the AIEOP-BFM ALL 2009 study, and 1/23 patients (4.3%) in the AIEOP-BFM ALL 2017 study), compared to the for the most part much higher prevalence reported in the literature. The patients who had deep vein thrombosis in our collective were on average 9.13 years old (range 3.27–17.9 years; Table 3). Interestingly, four (80%) out of the five patients with deep thrombosis were male. Risk factors for the development of thrombotic complications, such as hypertriglyceridemia, hyperleukocytosis, sepsis or adipositas, are not relevant here. Patient #4 in Table 3 had a heterozygous mutation of Factor II (G20210A) and a homozygous MTHFR mutation (C677T) as risk factors for developing thrombosis. Interestingly, only one of the patients with deep vein thrombosis did not require AT III administration, suggesting that there were no severe thromboses in the group of patients who did not require AT III administration.

**Table 3:** Overview of severe thrombotic complications despite prophylaxis with enoxaparin and AT III

Patients with deep vein thrombosis	Location of thrombosis	Risk factors (hypertriglyceridemia, hyperleukocytosis, sepsis, adipositas, genetic)	Gender	Age at thrombosis [years]	Treatment received for thrombosis	Interruption of L-asparaginase during further therapy	Recurrence of deep vein thrombosis at a later date
#1	V. axillaris/sub-clavia sinistra	no	male	7.35	Unfractionated heparin via perfusor for 5 days, then switch to enoxaparin twice daily in therapeutic dose for 12 days, after that once daily	no	no
#2	Sinus vein	no	female	3.27	Unfractionated heparin via perfusor for 5 days, then switch to enoxaparin twice daily in therapeutic dose until recanalization of sagittal sinus after 8 weeks	no	no
#3	Vv. iliacae internae	Massive hypertriglyceridemia, adipositas	male	12.03	Unfractionated heparin via perfusor (dose 20 IE/kg/h) for 14 days, then switch to enoxaparin twice daily in therapeutic dose	no	no
#4	V. fibularis sinistra	Heterozygous mutation FactorII (G20210A), homozygous MTHFR mutation (C677T)	male	17.90	Enoxaparin twice daily in therapeutic dose during whole intensive therapy, once daily during maintenance	no	no
#5	Vv. iliacae externae	no	male	5.11	Enoxaparin twice daily in therapeutic dose	no	no

## Discussion

Thromboembolic complications are well-known events in pediatric acute lymphoblastic leukemia patients and can lead to mortality and excess morbidity. They are strongly associated with administration of ASP and also glucocorticoids [11, 12]. The state of hypercoagulability may be attributed to hemostatic derangement with marked hypofibrinolysis [13] and decreased natural anticoagulants (AT III, protein C, protein S) [3, 14] associated with increased thrombin generation indicated by elevated D-dimer levels [4], whereby the drop in AT III is most strongly described in the literature [3]. In line with the literature, we were also able to show a marked decline in AT III (Fig. 1A) and fibrinogen (Fig. 1B) during the first 40 days of treatment, whereby the drop in AT III is not as evident as described in the literature since it was regularly substituted in patients with AT III levels below 50%. As stated above, it has been reported that thrombosis is more likely to occur during ASP therapeutic phase due to the relative procoagulant state caused by marked fibrinolysis inhibition. However, the present retrospective study cannot show clear data to suggest this point. If these points can be evaluated, the duration of anticoagulation therapy may change.

The incidence of thromboembolic complications under ALL therapy is reported differently in the literature: Nowak-Göttl et al. in 2009 described a symptomatic thrombosis prevalence of up to 36% in children with ALL during therapy with ASP and glucocorticoids [1]. Similar figures were published by Mitchell et al., who reported a prevalence of deep vein thrombosis of 36.7% in pediatric ALL patients [15]. Other authors reported an incidence of thromboembolic complications as 16.7% [4] or 6.2% [16] in pediatric ALL cohorts. As early as 2008, our department was able to demonstrate a 12.7% frequency of deep vein thrombosis in a historical cohort of 71 pediatric ALL patients, who were treated according to the AIEOP-BFM 95/2000 protocols [6]. A newer study from Austria [17] finally showed an incidence of thromboembolic events (≥ grade 2) of < 5%.

Regarding risk factors for developing thromboembolic complications during therapy, patients with ALL and ≥ 10.0 years of age who are treated according to the BFM regimen are particularly affected (compared to JACLS – Japan Association of Childhood Leukemia Study ALL-02 protocol) [13]. Adolescent age (10–16 or 10–18 years, retrospectively) was also reported by other authors to be a main risk factor for the development of thromboembolic events [17], as this patient cohort shows a more severe decline in anticoagulant and fibrinolytic parameters [18]. Furthermore, obese pediatric ALL patients showed a three-fold increased risk of developing a thromboembolic complication (symptomatic or asymptomatic) [19]. Parallel thereto, in this study, thrombelastography did not predict the development of thromboembolic events [19], which is in line with our observations (data not shown).

There is still conflicting evidence regarding thromboprophylaxis in children undergoing ALL treatment – comparable to the data available for adult ALL patients [20]. However, there is unanimous agreement that prophylactic use of anticoagulants should be considered in at least some patients during induction/consolidation. It has repeatedly been shown in the literature that the administration of fresh frozen plasma has no effect on the frequency of thrombosis under asparaginase therapy [21, 22]. Elhasid et al. showed 2001 in a pilot study that enoxaparin is a safe and possibly effective means of preventing thromboembolism in ALL patients during L-asparaginase therapy [23]. As early as 2008, we were able to show that a combination of enoxaparin and AT III is a safe and efficient option for thrombosis prophylaxis during ALL induction therapy [6]. In addition, Nowak-Göttl et al. mentioned a possible positive effect of AT III substitution even earlier [24]. A meta-analysis of six studies on the subject of thromboprophylaxis in children with cancer (especially ALL) showed that only the use of LMWH is safe and effective. A statement on AT III or vitamin K antagonists could not be made here [7]. A prospective study that examined the antithrombotic measures in the ALL-BFM 2000 and AIEOP-BFM ALL 2009 studies was able to show that the use of enoxaparin can be preferred to therapy with unfractionated heparin (thrombosis frequency 3.5% vs 8.0%). This study shows that the use of enoxaparin in children and adolescents with ALL in induction therapy can be recommended; the role of AT III remains to be determined [8]. Another prospective study has set itself the task of measuring the use of LMWH in comparison to no prophylaxis in children and adolescents with ALL in the Netherlands [9]. In addition, according to Pelland-Marcotte et al. [25], there is no evidence for a recommendation for antithrombotic prophylaxis solely because children have central catheters.

After 20 years of experience with consistently performed anti-thrombotic prophylaxis with LMWH and AT III substitution in all pediatric ALL patients in induction therapy, our patient population shows a very low incidence of deep venous thrombotic events (2.9%, Table 2). However, it should be mentioned here that in this study only symptomatic patients were examined for thrombosis using imaging. The fact that four of the five patients with deep vein thrombosis were male is interesting and could possibly be explained by the fact that male gender is also approximately 1.3 times overrepresented in the diagnosis of childhood leukemia. The need for a particular number and duration of AT III substitutions at serum values below 50% was independent of gender, but significantly dependent on age. We observed that adolescent patients had a greater decrease in AT III than did younger patients and therefore needed more AT III administration (total population *p* < 0.0001; female *p* < 0.0001; male *p* = 0.003; see also Table 2: need for AT III substitution: > 10 years of age 91.4% versus 65.2% in patients < 10 years of age). In addition, especially female adolescents showed these low AT III values for a longer time, which meant a longer period of AT III substitution (total population *p* = 0.064; female *p* = 0.022; male *p* = 0.567). This means that in the group of adolescents, prophylaxis with enoxaparin would be particularly important. Whether an additional substitution with AT III makes sense must be shown by large prospective studies. However, we have had good experience with a combination prophylaxis (enoxaparin and AT III) over the last 20 years, although it should always be kept in mind that increased AT III activity may decrease asparaginase activity [26].

## Conclusion

Thromboembolic complications are well known in the treatment of childhood acute lymphoblastic leukemia. Over the years, it has not been possible to reach a consensus on a possible prophylaxis for thromboembolic events. The only evidence in the literature shows the use of enoxaparin. From our 20 years of good experience with a combination therapy with enoxaparin and AT III substitution and based on our data, we can at least recommend it, especially from adolescence onwards.

## Data Availability

No datasets were generated or analysed during the current study.

## References

[1] Nowak-Göttl U, Kenet G, Mitchell LG (2009). Thrombosis in childhood acute lymphoblastic leukaemia: epidemiology, aetiology, diagnosis, prevention and treatment. Best Pract Res Clin Haematol.

[2] Mitchell L, Hoogendoorn H, Giles AR, Vegh P, Andrew M (1994). Increased endogenous thrombin generation in children with acute lymphoblastic leukemia: risk of thrombotic complications in L'Asparaginase-induced antithrombin III deficiency. Blood.

[3] Mitchell LG, Halton JM, Vegh PA, Barr RD, Venneri T, Pai KM (1994). Effect of disease and chemotherapy on hemostasis in children with acute lymphoid leukemia. Am J Pediatr Hematol Oncol.

[4] Ismail MM, Hamed GM (2017). Activity levels of natural anticoagulant proteins in childhood acute lymphoblastic leukemia: relation to thromboembolic complications and treatment. Blood Coagul Fibrinolysis.

[5] Biss TT, Payne JH, Hough RE, Grainger JD, Macartney C, Sibson KR (2016). Strategies to prevent and manage thrombotic complications of acute lymphoblastic leukemia in children and young people vary between centers in the United Kingdom. J Pediatr Hematol Oncol.

[6] Meister B, Kropshofer G, Klein-Franke A, Strasak AM, Hager J, Streif W (2008). Comparison of low-molecular-weight heparin and antithrombin versus antithrombin alone for the prevention of symptomatic venous thromboembolism in children with acute lymphoblastic leukemia. Pediatr Blood Cancer.

[7] Pelland-Marcotte MC, Tole S, Pechlivanoglou P, Brandão LR (2019). Effectiveness and safety of primary thromboprophylaxis in children with cancer: a systematic review of the literature and network meta-analysis. Thromb Haemost.

[8] Greiner J, Schrappe M, Claviez A, Zimmermann M, Niemeyer C, Kolb R (2019). THROMBOTECT - a randomized study comparing low molecular weight heparin, antithrombin and unfractionated heparin for thromboprophylaxis during induction therapy of acute lymphoblastic leukemia in children and adolescents. Haematologica.

[9] Klaassen ILM, Lauw MN, van de Wetering MD, Biemond BJ, Middeldorp S, Abbink FCH (2017). TropicALL study: thromboprophylaxis in children treated for acute lymphoblastic leukemia with low-molecular-weight heparin: a multicenter randomized controlled trial. BMC Pediatr.

[10] O'Brien SH, Li D, Mitchell LG, Hess T, Zee P, Yee DL (2019). PREVAPIX-ALL: apixaban compared to standard of care for prevention of venous thrombosis in paediatric Acute Lymphoblastic Leukaemia (ALL)-rationale and design. Thromb Haemost.

[11] Mall V, Thomas KB, Sauter S, Niemeyer CM, Sutor AH (1999). Effect of glucocorticoids, E. coli- and Erwinia L-asparaginase on hemostatic proteins in children with acute lymphoblastic leukemia. Klin Padiatr..

[12] Mitchell LG, Sutor AH, Andrew M (1995). Hemostasis in childhood acute lymphoblastic leukemia: coagulopathy induced by disease and treatment. Semin Thromb Hemost.

[13] Ishihara T, Nogami K, Ochi S, Ishida T, Kosaka Y, Sawada A (2020). Disordered hemostasis associated with severely depressed fibrinolysis demonstrated using a simultaneous thrombin and plasmin generation assay during L-asparaginase induction therapy in pediatric acute lymphoblastic leukemia. Pediatr Blood Cancer.

[14] Risseeuw-Appel IM, Dekker I, Hop WC, Hählen K (1994). Minimal effects of E. coli and Erwinia asparaginase on the coagulation system in childhood acute lymphoblastic leukemia: a randomized study. Med Pediatr Oncol..

[15] Mitchell LG, Andrew M, Hanna K, Abshire T, Halton J, Anderson R (2003). A prospective cohort study determining the prevalence of thrombotic events in children with acute lymphoblastic leukemia and a central venous line who are treated with L-asparaginase: results of the Prophylactic Antithrombin Replacement in Kids with Acute Lymphoblastic Leukemia Treated with Asparaginase (PARKAA) Study. Cancer.

[16] Ghanem KM, Dhayni RM, Al-Aridi C, Tarek N, Tamim H, Chan AKC, Saab R, Abboud MR, El-Solh H, Muwakkit SA. Cerebral sinus venous thrombosis during childhood acute lymphoblastic leukemia therapy: Risk factors and management. Pediatr Blood Cancer. 2017;64(12). 10.1002/pbc.26694. Epub 2017 Jun 29. 10.1002/pbc.26694 28660695

[17] Gidl A, Füreder A, Benesch M, Dworzak M, Engstler G, Jones N (2023). Incidence and risk factors of venous thromboembolism in childhood acute lymphoblastic leukaemia - a population-based analysis of the Austrian Berlin-Frankfurt-Münster (BFM) study group. Pediatr Hematol Oncol.

[18] Appel IM, Hop WC, van Kessel-Bakvis C, Stigter R, Pieters R (2008). L-Asparaginase and the effect of age on coagulation and fibrinolysis in childhood acute lymphoblastic leukemia. Thromb Haemost.

[19] Prasca S, Carmona R, Ji L, Ko RH, Bhojwani D, Rawlins YA (2018). Obesity and risk for venous thromboembolism from contemporary therapy for pediatric acute lymphoblastic leukemia. Thromb Res.

[20] Rank CU, Lynggaard LS, Als-Nielsen B, Stock W, Toft N, Nielsen OJ (2020). Prophylaxis of thromboembolism during therapy with asparaginase in adults with acute lymphoblastic leukaemia. Cochrane Database Syst Rev..

[21] Halton JM, Mitchell LG, Vegh P, Eves M, Andrew ME (1994). Fresh frozen plasma has no beneficial effect on the hemostatic system in children receiving L-asparaginase. Am J Hematol.

[22] Klaassen ILM, Zuurbier CCM, Hutten BA, van den Bos C, Schouten AYN, Stokhuijzen E (2019). Venous thrombosis in children with acute lymphoblastic leukemia treated on DCOG ALL-9 and ALL-10 protocols: the effect of fresh frozen plasma. TH Open.

[23] Elhasid R, Lanir N, Sharon R, Weyl Ben Arush M, Levin C, Postovsky S (2001). Prophylactic therapy with enoxaparin during L-asparaginase treatment in children with acute lymphoblastic leukemia. Blood Coagul Fibrinolysis..

[24] Nowak-Göttl U, Kuhn N, Wolff JE, Boos J, Kehrel B, Rath B (1996). Inhibition of hypercoagulation by antithrombin substitution in E. coli L-asparaginase-treated children. Eur J Haematol..

[25] Pelland-Marcotte MC, Amiri N, Avila ML, Brandão LR (2020). Low molecular weight heparin for prevention of central venous catheter-related thrombosis in children. Cochrane Database Syst Rev..

[26] Czogała M, Balwierz W, Sztefko K, Rogatko I (2017). Antithrombin III as the indicator of L-asparaginase activity in children treated for acute lymphoblastic leukemia. J Pediatr Hematol Oncol.

